# Preoperative contributing factors and the remission of diabetes after metabolic surgery: the mediating role of preoperative triglyceride

**DOI:** 10.1007/s40519-024-01647-7

**Published:** 2024-03-04

**Authors:** Lijuan Niu, Liqian Mu, Runda Wu, Shan Tong, Zhongqi Mao, Yi Yang, Jun Yin

**Affiliations:** 1 Department of General Surgery, The First Affiliated Hospital of Soochow University, Suzhou, Jiangsu China; 2 Department of Endocrinology, The First Affiliated Hospital of Soochow University, Suzhou, 215000 Jiangsu China; 3 Department of Neurology, The First Affiliated Hospital of Soochow University, Suzhou, Jiangsu China

**Keywords:** Diabetes mellitus, Obesity, Metabolic surgery, Mediating role, Preoperative triglyceride

## Abstract

**Background and objective:**

Limited understanding exists regarding the factors affecting the prognosis of surgical treatment for type 2 diabetes mellitus (T2DM), particularly in Chinese patients. In this study, we examined a cohort of early and intermediate obese T2DM patients to explore the potential impact of preoperative lipid metabolism in metabolic surgery on the postoperative remission of T2DM.

**Methods:**

Participants with T2DM and obesity underwent metabolic surgery. Clinical data, including baseline body mass index, percentage of excess weight loss, and preoperative biochemical indicators, were collected and analyzed. A multidisciplinary team conducted patient follow-up. Remission was defined as sub-diabetic hyperglycemia (HbA1c < 48 mmol/mol, fasting glucose 100–125 mg/dl) without pharmacological intervention for at least 12 months.

**Results:**

Over a median follow-up of 27 months, 96 T2DM patients with metabolic surgery were studied, with no laparotomies required. Among these patients, 61 (63.5%) achieved complete remission, and 85 (88.5%) experienced remission. In multivariable analysis models, preoperative fasting blood glucose (FBG) significantly correlated with all postoperative outcomes. Furthermore, mediation analysis indicated that preoperative triglycerides (TG) mediated 26.31% of the association between preoperative FBG and postoperative remission. Both preoperative FBG and TG were negatively associated with the postoperative remission of T2DM.

**Conclusion:**

In summary, our findings suggest that lower preoperative fasting glucose levels enhance the likelihood of postoperative T2DM remission. Moreover, preoperative TG could potentially play a mediating role in the postoperative remission of T2DM. Therefore, evaluating and managing fasting glucose and lipids before the procedure may aid in assessing the prognosis of metabolic surgery.

*Level of evidence* Level III, designed cohort.

## Introduction

As a traditionally intractable chronic medical condition, the medical management of type 2 diabetes mellitus (T2DM) typically consists of lifestyle modifications and specific glucose-lowering medication [1, 2]. Conservative approaches provide short-term benefits for most patients, but rarely lead to persistent clinical remission or metabolic improvement [3, 4]. Additionally, the pharmacotherapy of T2DM often focuses solely on managing hyperglycemia, rather than addressing the various metabolic disorders associated with the disease [4]. The pathogenesis of insulin resistance and T2DM is intricately linked to the presence of overall and visceral adiposity [5, 6]. Recently, metabolic surgery has emerged as a notable therapeutic approach that has garnered considerable attention due to its potential for ameliorating metabolic dysregulation and serving as a treatment modality for T2DM [7]. In a large-scale study, a significant proportion of patients (75%) achieved remission of T2DM two years following metabolic surgery [8]. Moreover, this remission was accompanied by substantial weight loss and demonstrated favorable outcomes, including a regression of microalbuminuria and a reduced incidence of vascular complications [9]. These findings underscore the notable metabolic benefits of metabolic surgery, highlighting its potential to not only address T2DM, but also improve associated metabolic disorders and mitigate the risk of complications.

However, the long-term outcomes of metabolic surgery remain uncertain. Meanwhile, lipid metabolism, particularly fasting triglyceride levels, has been established as a significant factor in insulin resistance and diabetes [10]. Furthermore, its presence in individuals with prediabetes serves as a crucial predictor for the development of diabetes [11]. Therefore, in the context of metabolic surgery, is there a correlation between lipid metabolism and the remission of T2DM? The existing data on this topic are still limited. In summary, considering the significance of lipid metabolism, particularly triglyceride levels, in the pathogenesis and management of diabetes, we propose a hypothesis that preoperative lipid metabolism might impact the remission of diabetes following metabolic surgery.

In this cohort study, we gathered demographic and clinical data from obese patients with T2DM who underwent metabolic surgery. Our aim was to examine the association between preoperative indicators and prognosis from various perspectives during the follow-up period. Additionally, we sought to investigate whether the mediating role of preoperative lipid metabolism, particularly triglyceride levels in the association between metabolic surgery and postoperative remission of diabetes. This research aims to enhance our understanding of patient selection and prognosis in the context of metabolic surgery, with the ultimate goal of improving outcomes for individuals undergoing this procedure. Meanwhile, these insights are crucial for understanding the pathophysiological underpinnings of impaired glucose homeostasis and may pave the way for targeted therapeutic interventions in the management of metabolic disorders.

## Methods

### Study population

We conducted a single-cohort study on individuals with T2DM who underwent metabolic surgery at the First Affiliated Hospital of Soochow University between January 2013 and July 2018. T2DM was defined as (1) using diabetes medication; (2) fasting whole blood glucose ≥ 7.0 mmol/L and/or random whole blood glucose ≥ 11.1 mmol/L; (3) patients according to national or local patient registers with diabetes [12]. The diagnosis and classification of T2DM are based on the criteria established by the American Diabetes Association [13]. Diabetic patients were recruited from outpatient and emergency clinics and managed by a multidisciplinary team, which included endocrinologists and general surgeons. All participants underwent nutritional, psychological, and endocrinological assessments.

**Fig. 1:**
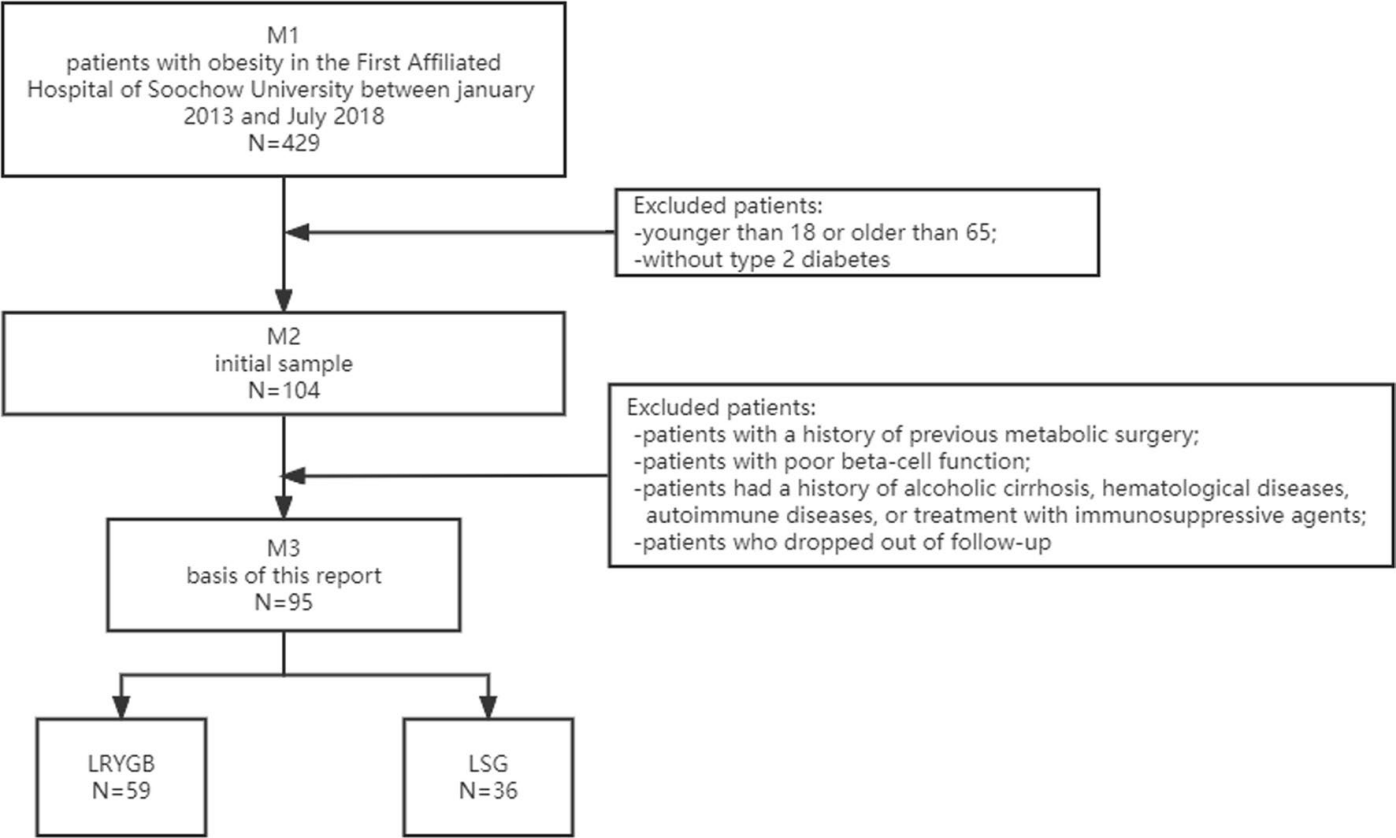
Flowchart

Inclusion criteria were as follows: (1) patients aged between 18 and 65 years; (2) patients without previous history of metabolic surgery; (3) non-type 1 diabetic patients with adequate beta-cell function (excluding those diagnosed with anti-GAD or islet cell autoantibodies, individuals with over ten years of insulin use, fasting C-peptide levels below 1 ng/mL, or those unresponsive to stimulation tests)[14]; (4) patients with no history of alcoholic liver cirrhosis, hematologic disease, autoimmune disease, or history of receiving immunosuppressive therapy; (5) patients with body mass index (BMI) > 32 kg/m^2^ and poorly controlled T2DM after 6 months of nutritional intervention and glucose-lowering therapy (Fig. 1).

Following the operation, all patients were monitored by a case manager and received consistent medical advice and treatment, including a well-balanced diet and effective blood pressure control.

### Surgical procedures

The surgical procedures consisted of two types of metabolic procedures: (1) laparoscopic Roux-en-Y gastric bypass (LRYGB); (2) laparoscopic sleeve gastrectomy (LSG). The selection of the procedure was based on patients' preferences following a comprehensive preoperative conference with the multidisciplinary team. All procedures were performed by the same experienced surgical team. Briefly, we used a standard 5-port laparoscopic technique. The LRYGB operation involves an antecolic, antegastric Roux limb, a 100-cm biliopancreatic limb, and a 100-cm alimentary limb. The gastric pouch was approximately 30 mL, and the gastrojejunostomy was created by a stapler technique with an anastomosis 1.0–1.5 cm in diameter. LSG was performed by creating a sleeve gastrectomy over a 36Fr bougie and leaving a 4–6 cm long antrum. Thromboembolic prophylaxis consisted of perioperative pneumatic compression and low-molecular-weight heparin (4000AxaIU) during anesthetic induction.

### Clinical information collection

Demographics were collected through electronic patient records and administrative databases. The patients’ body weight was measured while wearing light clothing and without shoes, rounded to the nearest 0.1 kg, and their body height was measured to the nearest 0.1 cm. BMI was calculated by dividing the weight in kilograms by the height in meters squared. Preoperatively, complete blood cell counts and blood biochemical parameters, including the individual components of glycemic control such as serum glucose levels and glycated hemoglobin (HbA1c) levels, were assessed. Peripheral venous blood samples were collected on the morning of the second day after admission following an overnight fasting period.

### Outcome variables

The observation period was 13 to 36 months. The endpoint was the complete remission of T2DM. Several secondary outcome measures were assessed, including partial remission of T2DM and the postoperative percentage of excess weight loss (%EWL) at 6 and 12 months. A good outcome was defined as a %EWL of over 50% [15]. Complete remission was defined as achieving glycemia below the diabetic range (HbA1c in the normal range [< 6.0%], fasting glucose < 5.6 mmol/L) without pharmacological intervention for at least 12 months [16]. Partial remission was defined as sub-diabetic hyperglycemia (HbA1C not diagnostic of diabetes [< 6.5%], fasting glucose 100–125 mg/dl [5.6–6.9 mmol/L]) without pharmacological intervention for at least 12 months [16]. The rationale behind selecting these specific variables is rooted in their relevance to the comprehensive evaluation of the effectiveness of metabolic surgery in managing T2DM.

The initial clinical evaluation took place one week after discharge. Subsequently, postoperative clinical and laboratory evaluations, including fasting glucose, glycosylated hemoglobin, and blood count, were conducted on a monthly basis. These assessments contribute to a comprehensive and ongoing analysis of the surgical intervention’s efficacy in achieving and maintaining glycemic control, thereby informing the study’s overarching objectives.

### Data analyses

For descriptive purposes, the differences among continuous variables were analyzed by the Student’s t-test or the Mann–Whitney U test, while the Chi-square test assessed differences among categorical variables. Pearson’s correlation coefficients were calculated to assess the relationship between the variables. Logistic regression analysis was used to find contributing factors associated with remission of T2DM in patients receiving metabolic surgery. Models were adjusted for gender. Additionally, the level of significance for these descriptive comparisons was established at 0.05 for two-sided hypothesis testing.

Subsequently, we performed a mediating effect analysis based on the bootstrap method proposed by Preacher and Hayes through R-packet ‘mediating’ to explore whether preoperative lipid metabolism, particularly triglyceride levels could be modes or mechanisms by which metabolic surgery influences on the remission of diabetes [17]. Statistical analysis was performed in SPSS 26.0, R programming language 4.3.0, and GraphPad Prism 9.3.0.

## Results

### Participants and descriptive characteristics

The initial sample included 429 patients, out of which only 104 individuals with obesity and a diagnosis of T2DM were recruited for the study. The median observation time was 27 months with ranges between 14 and 37 months. Eight patients were lost (9/104, 8.7%) and 95 participants (59 LRYGB and 36 LSG, no conversion to laparotomy) with at least 1 year of follow-up finally formed the basis of this report. Only 1 patient was reported to have an anastomotic leak; no surgical mortality during the follow-up. As the number of participants with severe postoperative complications and death was very small, no useful separate analysis could be made.

Among all subjects, 46.3% were males (44/95); the age was 35.0 (30.0, 45.0) years old; duration of DM was 6 (4, 9) years; according to the ultrasound results, 92 percent of the patients had mild-to-moderate fatty liver and 8 percent had severe fatty liver; 60 (63.2%) patients had complete remission; and 11 (11.6%) patients did not achieve postoperative remission. Meanwhile, %EWL at postoperative 6 months was 66.7 (58.4, 73.2); %EWL at postoperative 12 months was 72.9 (67.3, 81.0). Patient characteristics are shown in Table 1.

**Table 1:** The characteristics of patients receiving surgical T2DM treatment

Characteristics	Patients (*N* = 95)
Demographics and medical history	
Age in years, median (IQR)	35.0 (30.0, 45.0)
Male, *n* (%)	44 (46.3)
Smoking status, *n* (%)	28 (29.5)
Alcohol consumption, *n* (%)	20 (21.1)
Duration of DM in year, median (IQR)	6 (4, 9)
Insulin usage, *n* (%)	11 (11.5)
Hypertension, *n* (%)	82 (86.3)
Stroke history, *n* (%)	6 (6.3)
Hyperlipidemia, *n* (%)	79 (83.2)
Clinical features	
Surgical procedure	
LRYGB, *n* (%)	59 (62.1)
LSG, *n* (%)	36 (37.9)
Follow-up time in month, median (IQR)	27 (20, 32)
Preoperative HbA1c (%), median (IQR)	7.9 (7.0, 10.4)
Preoperative C-peptide in ng/mL, median (IQR)	8.9 (5.3, 11.6)
Preoperative waist circumference in cm, median (IQR)	130 (124, 135)
Preoperative BMI, median (IQR)	39.2 (34.4, 44.3)
Preoperative FBG in mmol/L, median (IQR)	8.2 (6.4, 10.8)
Preoperative HbA1c (%), median (IQR)	
Preoperative WBC in × 10^9^ /L, median (IQR)	8.0 (6.7, 9.2)
Preoperative N in × 10^9^ /L, median (IQR)	4.7 (3.5, 5.7)
Preoperative L in × 10^9^ /L, median (IQR)	2.5 (2.0, 2.9)
Preoperative M in × 10^9^ /L, median (IQR)	0.45 (0.4, 0.6)
Preoperative platelets in × 10^9^ /L, median (IQR)	237.0 (194.0, 275.0)
Preoperative RDW (%), median (IQR)	12.9 (12.6, 13.6)
Preoperative creatinine in μmol/L, median (IQR)	54.0 (46.0, 66.3)
Preoperative TC in mmol/L, mean ± SD	4.7 ± 1.0
Preoperative TG in mmol/L, median (IQR)	1.97 (1.4, 3.0)
Outcomes	
Complete remission, *n* (%)	60 (63.2)
Remission (partial or complete), *n* (%)	84 (88.4)
%EWL at 6 months after operation, median (IQR)	66.7 (58.4, 73.2)
%EWL at 12 months after operation, median (IQR)	72.9 (67.3, 81.0)

*IQR* interquartile range, *BMI* body mass index, *FGB* fasting blood glucose, *TC* total cholesterol, *TG* triglyceride, *%EWL* percentage excess weight loss

### Comparison of clinical characteristics in patients receiving metabolic surgery

According to the complete remission of T2DM or not, these participants were divided into two groups: the complete remission group with 60 patients and the non-complete remission group with 35 patients. Statistical analysis indicated that there were significant differences in stroke history, preoperative HbA1c, preoperative C-peptide and preoperative fasting blood glucose (FBG) (*p* < 0.05). However, there was no difference in baseline total cholesterol (TC), triglyceride (TG), BMI or other factors between two groups (*p* > 0.05, Table 2). Meantime, according to the remission of T2DM or not, these participants were divided into two groups: the remission group with 84 patients and the non-remission group with 11 patients. Statistical analysis indicated that there were significant differences in preoperative HbA1c, preoperative FBG and TG (*p* < 0.05). However, there was no difference in other factors between two groups (*p* > 0.05, Table 3).

**Table 2:** Comparison of descriptive characteristics between groups of patients with complete remission or not

Characteristics	Complete remission (*N* = 60)	Non-complete remission (*N* = 35)	*P value*
Demographics and medical history			
Age in years, median (IQR)	36.0 (28.8, 46.3)	35.0 (30.5, 42.0)	0.932
Male, *n* (%)	33 (55.0)	18(51.4)	0.902
Smoking status, *n* (%)	17 (28.3)	11 (31.4)	0.932
Alcohol consumption, *n* (%)	12 (20.0)	8 (22.9)	0.945
Duration of DM in month, median (IQR)	6 (4, 8)	6 (4, 9)	0.997
Insulin usage, *n* (%)	7 (11.4)	4(11.4)	1.000
Hypertension, *n* (%)	52 (86.7)	30 (85.7)	1.000
Stroke history, *n* (%)	1 (1.7)	5 (14.3)	0.024
Hyperlipidemia, *n* (%)	49 (81.7)	30 (85.7)	0.822
Clinical features			
Surgical procedure			
LRYGB, *n* (%)	34 (56.7)	25 (71.4)	0.226
LSG, *n* (%)	26 (43.3)	10 (28.6)	
Preoperative HbA1c (%), median (IQR)	10.2 ± 4.8	7.3 ± 4.4	0.013
Preoperative C-peptide in ng/mL, mean ± SD	7.4 (6.5, 8.5)	10 (8.5, 11.5)	< 0.001
Preoperative BMI, median (IQR)	39.4 (35.1, 44.8)	39.1 (33.7, 42.4)	0.298
Preoperative FBG in mmol/L, median (IQR)	6.8 (5.6, 8.7)	11.2 (9.0, 13.4)	< 0.001
Preoperative WBC in × 10^9^ /L, median (IQR)	8.3 (6.5, 9.9)	7.5 (6.7, 8.7)	0.079
Preoperative N in × 10^9^ /L, median (IQR)	5.0 (3.6, 6.1)	4.6 (3.4, 5.2)	0.111
Preoperative L in × 10^9^ /L, median (IQR)	2.5 (2.0, 2.9)	2.4 (2.0, 2.9)	0.522
Preoperative M in × 10^9^ /L, median (IQR)	0.5 (0.4, 0.6)	0.4 (0.4, 0.6)	0.610
Preoperative creatinine in μmol/L, median (IQR)	56.9 (46.0, 69.0)	52.0 (46.0, 60.0)	0.128
Preoperative TC in mmol/L, mean ± SD	4.7 ± 1.0	4.9 ± 1.1	0.326
Preoperative TG in mmol/L, median (IQR)	1.9 (1.4, 2.8)	2.3 (1.7, 3.5)	0.059

*IQR* interquartile range, *BMI* body mass index, *FBG* fasting blood glucose, *TC* total cholesterol, *TG* triglyceride, *%EWL* percentage excess weight loss

**Table 3:** Comparison of descriptive characteristics between groups of patients with remission (partial or complete) or not

Characteristics	Remission (*N* = 84)	Non-remission (*N* = 11)	*P value*
Demographics and medical history			
Age in years, median (IQR)	35.0 (29.0, 45.3)	37.0 (31.0, 40.0)	0.789
Male, *n* (%)	45 (53.6)	6 (54.5)	1.000
Smoking status, *n* (%)	27(32.1)	1 (9.1)	0.166
Alcohol consumption, *n* (%)	19 (22.6)	1 (9.1)	0.448
Duration of DM in month, median (IQR)	6 (4,8)	6 (4, 14)	0.892
Insulin usage, *n* (%)	8 (9.52)	3(27.27)	0.452
Hypertension, *n* (%)	73 (86.9)	9(81.8)	0.644
Stroke history, *n* (%)	4 (4.8)	2 (18.2)	0.142
Hyperlipidemia, *n* (%)	70 (83.3)	9 (81.8)	1.000
Clinical features			
Surgical procedure			
LRYGB, *n* (%)	51 (60.7)	8 (72.7)	0.525
LSG, *n* (%)	33 (39.3)	3 (27.3)	
Preoperative HbA1c (%), median (IQR)	7.6 (6.8, 9.9)	10.6 (9.3, 11.2)	0.003
Preoperative C-peptide in ng/mL, mean ± SD	8.9 (5.4, 12.0)	7.1 (3.4, 11.1)	0.291
Preoperative BMI, median (IQR)	39.5 (34.6, 44.8)	34.9 (32.8, 41.0)	0.100
Preoperative FBG in mmol/L, median (IQR)	7.5(5.9, 9.8)	11.5 (10.9, 12.4)	0.001
Preoperative WBC in × 10^9^ /L, mean ± SD	8.0 (6.7, 9.3)	7.5 (6.5, 8.6)	0.269
Preoperative N in × 10^9^ /L, median (IQR)	4.7 (3.5, 5.9)	4.9 (3.4, 5.3)	0.432
Preoperative L in × 10^9^ /L, median (IQR)	2.5 (2.0, 2.9)	2.35 (1.9, 2.8)	0.396
Preoperative M in × 10^9^ /L, median (IQR)	0.5 (0.4, 0.6)	0.4 (0.3, 0.5)	0.247
Preoperative creatinine in μmol/L, median (IQR)	54.2(46.0, 69.6)	49.0 (46.5, 61.5)	0.727
Preoperative TC in mmol/L, mean ± SD	4.7 ± 0.9	4.7 ± 1.3	0.952
Preoperative TG in mmol/L, median (IQR)	1.9 (1.4, 2.8)	3.83 (1.7, 9.0)	0.024

*IQR* interquartile range, *BMI* body mass index, *FBG* fasting blood glucose, *TC* total cholesterol, *TG* triglyceride, *%EWL* percentage excess weight loss

**Fig. 2:**
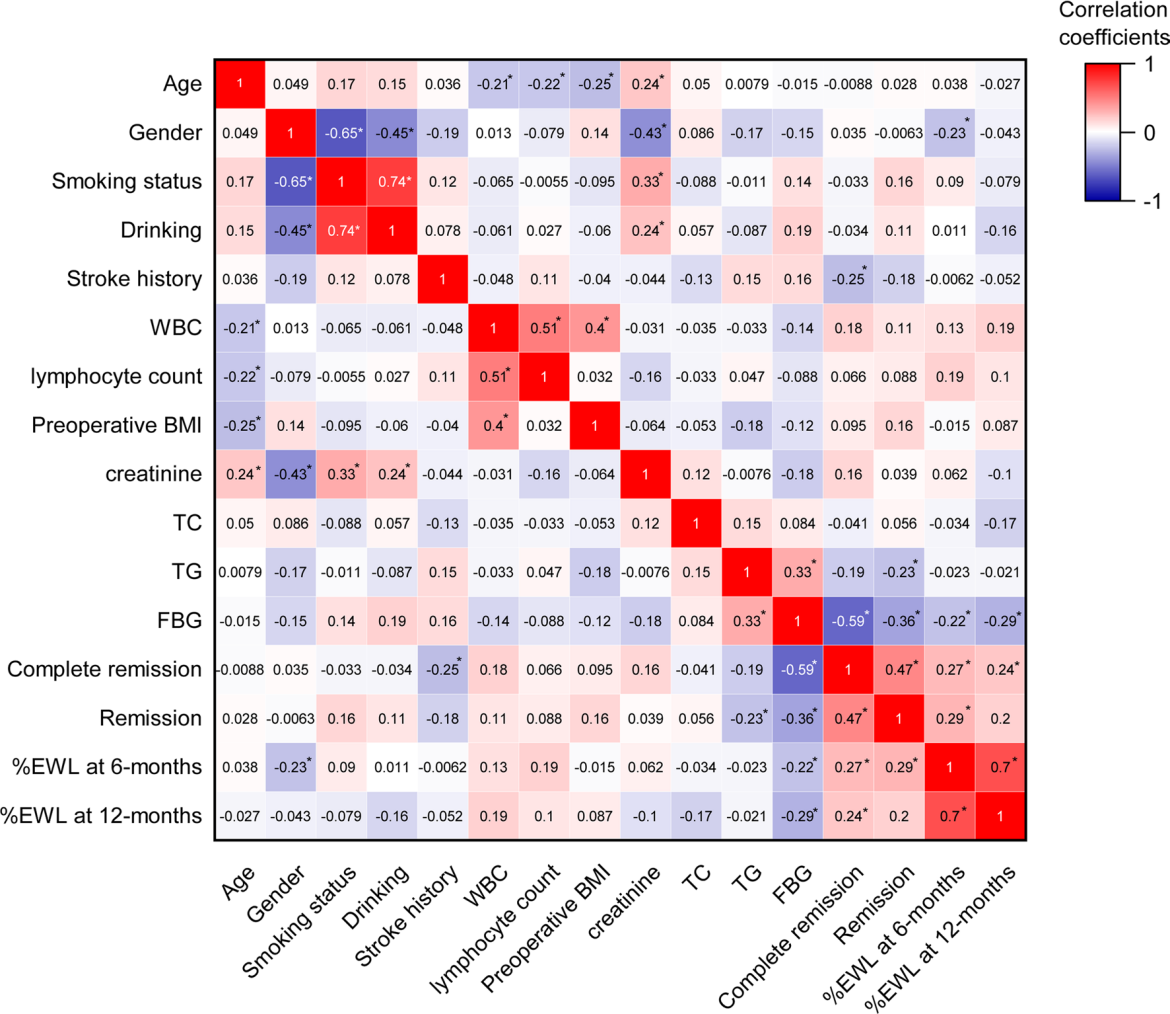
The heat map for Pearson correlation coefficients between the variables

Furthermore, we calculated the correlation coefficients between age, gender, smoking, drinking, hypertension, coronary disease, stroke history, BMI, blood biochemical criterion, complete remission, partial remission, postoperative %EWL at 6 months, and 12 months. Observed correlation coefficients: *r* = 0.33 between preoperative TG and FBG (*p* < 0.05); *r* = 0.23 between preoperative TG and remission (*p* < 0.05). Postoperative remission had weak but significant correlations with preoperative TG and preoperative FBG correlated meaningfully with all postoperative outcomes (Fig. 2).

### Contributing factors associated with prognosis in postoperative T2DM patients

Multivariable logistic regression analyses were used to identify the risk factors associated with prognosis in postoperative T2DM patients. Considering the correlation among smoking status, alcohol consumption, and gender in China (tend to be overwhelmingly male; Table 4), we did not bring them into the adjustment factors. For the overall sample, we found that the preoperative FBG was significantly associated with the complete postoperative remission of T2DM (OR: 0.566, 95% CI: 0.446–0.719), and the remission (partial or complete) of T2DM (OR: 0.785, 95% CI: 0.664–0.928), and the %EWL at postoperative 6 months (OR: 0.776, 95% CI: 0.658–0.917), and the %EWL at postoperative 12 months (OR: 0.761, 95% CI: 0.638–0.908). Additionally, preoperative WBC was associated with the complete postoperative remission of T2DM (OR: 1.263, 95% CI: 1.003–1.591) (Table 4).

**Table 4:** Logistic regression analysis of contributing factors associated with prognosis in patients receiving surgical T2DM treatment

Factor	Total (*N* = 95)	OR (95% CI)
Complete remission	60 (63.2)	
Preoperative FBG in mmol/L		0.566 (0.446, 0.719)*
Preoperative WBC in × 10^9^/L		1.263 (1.003, 1.591)*
Preoperative creatinine in μmol/L		1.013 (0.986, 1.04)
Preoperative platelets in × 10^9^/L		1.002 (0.996, 1.008)
Preoperative BMI		1.032 (0.967, 1.101)
Remission (partial or complete)	84 (88.4)	
Preoperative FBG in mmol/L		0.785 (0.664, 0.928)^*^
Preoperative WBC in × 10^9^/L		1.244 (0.873, 1.772)
Preoperative creatinine in μmol/L		0.984 (0.954, 1.015)
Preoperative platelets in × 10^9^/L		1.004 (0.995, 1.014)
Preoperative BMI		1.114 (0.982, 1.265)
%EWL at 6 months	80 (84.2)	
Preoperative FBG in mmol/L		0.776 (0.658, 0.917)*
Preoperative WBC in × 10^9^/L		1.216 (0.891, 1.659)
Preoperative creatinine in μmol/L		0.993 (0.962, 1.025)
Preoperative platelets in × 10^9^/L		0.996 (0.988, 1.004)
Preoperative BMI		1.024 (0.942, 1.112)
%EWL at 12 months	85 (89.5)	
Preoperative FBG in mmol/L		0.761(0.638, 0.908)^*^
Preoperative WBC in × 10^9^ /L		1.518 (0.99, 2.326)
Preoperative creatinine in μmol/L		0.980 (0.95, 1.012)
Preoperative platelets in × 10^9^ /L		1.004 (0.995, 1.014)
Preoperative BMI		1.070 (0.953, 1.201)

*p < 0.05

*IQR* interquartile range, *BMI* body mass index, *FGB* fasting blood glucose, *TC* total cholesterol, *TG* triglyceride, *%EWL* percentage excess weight loss. Additionally adjusted for gender

### Mediating role of preoperative TG

**Fig. 3:**
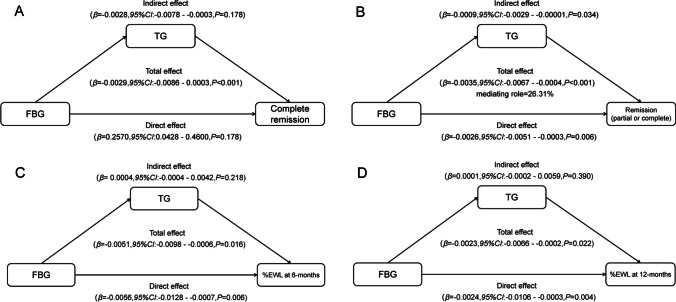
Mediated association between preoperative fasting blood glucose and the prognosis for metabolic surgery by triglyceride

A mediation analysis was performed to investigate whether preoperative TG mediates the association between preoperative FBG and prognosis in postoperative T2DM patients (Fig. 3).

The indirect associations via average preoperative TG implied that we would observe a − 0.0009-point (95% CI, − 0.0029 to − 0.00001) increase in the possibilities of postoperative remission among participants with T2DM. The proportion of the association between preoperative FBG and postoperative remission mediated by preoperative TG was 26.31%. However, we have not yet found that preoperative TG plays a significant mediating effect in the complete postoperative remission of T2DM and postoperative %EWL. In our further data analysis, we observed a positive correlation between preoperative FBG levels and serum TG. Additionally, both preoperative FBG and TG showed a negative association with the postoperative remission of T2DM.

## Discussion

In this study, we conducted an analysis of the surgical prognosis and preoperative blood biochemical indices in obese patients with T2DM. Our findings revealed that patients with lower preoperative fasting glucose levels exhibited a higher likelihood of experiencing postoperative remission of T2DM. Moreover, we observed that preoperative TG played a mediating role in the postoperative remission of T2DM, indicating a potential association between the prognosis of this procedure and the preoperative lipid metabolism profile. To the best of our knowledge, this study represents the first exploration of the role of preoperative blood biochemical indices, especially TG, as potential mediators in the postoperative remission of T2DM within the context of metabolic surgery. Meanwhile, the extended 13- to 36-month observation period provides a thorough understanding of metabolic surgery's long-term effects on T2DM remission. These strengths collectively underscore the robustness of our findings.

Considering the short and long-term challenges of metabolic surgery itself, the selection of patients and the assessment of preoperative indices become extremely important [18]. The progression from obesity to diabetes mellitus is a continuum that spans various stages, wherein defects in insulin resistance and insulin secretion play a crucial role in their interaction [19]. Excessive insulin secretion is recognized as an independent risk factor for T2DM [20]. Meanwhile, basal insulin hypersecretion is observed in obese populations even in the absence of insulin resistance, which is correlated with increased plasma lipid [21, 22]. The results of a recent study highlighted that better preoperative glycemic management promoted diabetic remission in postoperative patients [23]. Consistently, our findings indicate that patients with lower preoperative fasting glucose levels have a greater likelihood of experiencing postoperative T2DM remission. This observation may be attributed to the better preservation of pancreatic islet function in these individuals. These results align with the existing preoperative evaluation criteria for patients undergoing metabolic surgery [24].

The underlying mechanism for diabetes remission after metabolic surgery is intriguing, and weight loss is the fundamental component of this treatment, even for patients who are not severely obese [14, 18]. Furthermore, previous studies have demonstrated significant improvement in all diabetes-related markers following surgery [25]. However, it is noteworthy that these improvements were not correlated with the degree of weight loss [25]. The effects on glucose metabolism and insulin sensitivity do not seem to be solely reliant on weight loss. The postoperative enhancement in insulin resistance was rapid and independent of EWL, implying the involvement of other mechanisms in these alterations [25]. In populations with metabolic abnormalities, lipid metabolism and glucose homeostasis are closely intertwined, forming a complex interrelationship [26]. In obese individuals, basal insulin overproduction can lead to an increase in plasma lipids [26]. Moreover, in patients with normal glucose tolerance, dyslipidemia is strongly associated with pancreatic β-cell dysfunction, especially evident in those with elevated TC and LDL-C levels [27]. This observation may elucidate the negative correlation between preoperative TG levels and postoperative remission of T2DM in our analysis.

Excessive secretion of triglyceride-rich lipoproteins from the liver is a prominent characteristic of obesity when it is linked to metabolic syndrome [28]. Metabolic surgery has been shown to decrease the inflow of free fatty acids into the liver, consequently reducing the liver's production of triglyceride-rich lipoproteins [29]. In our data analysis, preoperative TG was found to mediate the negative association between preoperative FBG and postoperative T2DM remission. In muscle and liver, the accumulation of intracellular fat, specifically glycerol diacyls, leads to impairment of the insulin signaling pathway [30].

The deleterious effects of chronically elevated free fatty acid levels on glucose homeostasis are known as lipotoxicity, and concurrent exposure to high glucose may result in synergistic glycolipotoxicity [31]. This dual insult has adverse effects on various facets of insulin production and secretion processes [31]. The underlying molecular mechanisms include endoplasmic reticulum stress, oxidative stress, mitochondrial dysfunction, impaired autophagy, inflammation, etc.; the intricate crosstalk between these molecular pathways and stress factors occurs at multiple levels [32].

On the other hand, recent studies suggest that glucose and free fatty acids exhibit cooperative relationships in new research models, rather than competitive ones [33]. The free fatty acid acts as a buffer, thereby contributing to the maintenance of glucose homeostasis [33]. This suggests that lipid metabolism is deeply involved in the metabolic syndrome as well as in the entire pathogenesis of diabetes, pointing to a complex interrelationship between the two that maybe more intricate than previously hypothesized. Hence, it raises the question of whether stricter preoperative lipid management might also be beneficial for postoperative remission. Larger cohort studies could potentially offer additional insights and answers to this query.

### Strength and limits

Despite some previous studies have hinted at connections between glucose levels, triglycerides (TG), and the remission of type 2 diabetes (T2DM), our research injects novel dimensions into this field. More precisely, our study makes a distinctive contribution to the current body of literature by delving into previously unexplored variables or pathways associated with T2DM remission post-metabolic surgery, thereby enhancing our understanding of the multifaceted factors that influence postoperative outcomes.

Our data should be interpreted with some caution due to the limitations of the study. Since the participants in this study were recruited only from one clinical unit, there may have retrospective bias inherent due to the insufficient sample size. We did not assess lipid metabolism at follow-up time points. Additionally, being an observational study, we did not delve into the corresponding mechanisms of action of triglycerides before and after the entire metabolic surgery. As such, specific clinical implications necessitate further investigation.

### What is already known on this subject?

Metabolic surgery has demonstrated efficacy in ameliorating metabolic dysregulation and providing short-term relief for individuals with type 2 diabetes. Meanwhile, lipid metabolism, particularly fasting triglyceride levels, has been established as a significant factor in insulin resistance and diabetes.

### What this study adds?

Our study represents the first exploration of the role of preoperative blood biochemical indices, especially TG, as potential mediators in the postoperative remission of T2DM within the context of metabolic surgery. Meanwhile, the extended 13- to 36-month observation period provides a thorough understanding of metabolic surgery’s long-term effects on T2DM remission.

## Conclusion

In summary, our data suggest that most patients with T2DM benefit from metabolic surgery. Patients with lower preoperative fasting glucose levels have a higher likelihood of postoperative T2DM remission. Furthermore, our observations highlight the mediating role of preoperative triglyceride levels in the postoperative remission of T2DM. These findings contribute novel insights to the intricate interplay between fat metabolism and glucose homeostasis. Preoperative assessment and management of fasting glucose and lipids may be helpful in evaluating the prognosis of metabolic surgery and avoiding unnecessary procedures.

## Data Availability

The datasets used and/or analyzed during the current study are available from the corresponding author on reasonable request.

## Abbreviations

T2DM: Type 2 diabetes mellitus
BMI: Body mass index
LRYGB: Roux-en-Y gastric bypass
LSG: Laparoscopic sleeve gastrectomy
%EWL: Percentage excess weight loss
IQR: Interquartile range
TC: Total cholesterol
TG: Triglyceride
WBC: White blood cell
FBG: Fasting blood glucose
